# Influence of emotional complexity on the neural substrates of affective theory of mind

**DOI:** 10.1002/hbm.24794

**Published:** 2019-09-30

**Authors:** Marie Caillaud, Alexandre Bejanin, Mickael Laisney, Pierre Gagnepain, Malo Gaubert, Armelle Viard, Patrice Clochon, Vincent de La Sayette, Philippe Allain, Francis Eustache, Béatrice Desgranges

**Affiliations:** ^1^ Laboratoire de Psychologie des Pays de la Loire (LPPL), EA 4638 49100 Angers France; ^2^ Normandie Univ, UNICAEN, PSL Universités Paris, EPHE, INSERM, U1077, CHU de Caen, GIP Cyceron Caen France

**Keywords:** basic emotions, fMRI, self‐conscious emotions, temporoparietal junction, theory of mind

## Abstract

Affective theory of mind (ToM) depends on both the decoding of emotional expressions and the reasoning on emotional mental states from social situations. While previous studies characterized the neural substrates underlying these processes, it remains unclear whether the nature of the emotional state inferred from others can influence the brain activation associated with affective ToM. In the present study, we focused on two types of emotions: basic emotions (BEs) (e.g., anger and surprise), which are innate and universal and self‐conscious emotions (e.g., pride and embarrassment), which correspond to a special class of emotions involving the self and including a representation of one's relative reactions to internal and external standards. Specifically, we used an ecological functional MRI paradigm, on 21 healthy young subjects, to compare brain activations during the decoding of and the reasoning on others' self‐conscious, basic and neutral mental states. Our results showed that compared to neutral states, the inference of self‐conscious and basic emotional states from others elicited more activation in several core regions of affective ToM. Direct comparisons between emotional conditions revealed more activation for self‐conscious than BEs in the right temporoparietal junction during the reasoning process and in left middle occipital regions during the decoding process. Further analyses using a localizer task showed that the extrastriate body area was more recruited for decoding others' self‐conscious versus BEs, which emphasize the importance of body clues to properly infer these emotions. Using an original task allowing for an ecological assessment of the affective ToM, these results demonstrate that the complexity of the emotion inferred to others can influence the recruitment of ToM network. This study also validates the use of our task as an ecological tool to assess the affective ToM, constituting an avenue for the characterization of ToM impairments in neurological conditions.

## 1 INTRODUCTION

Affective theory of mind (ToM), also known as cognitive empathy according to some authors, is a complex cognitive function that enables one to infer the emotional states of other people and thus to anticipate and interpret their behaviors (Abu‐Akel & Shamay‐Tsoory, 2011). This ability is sustained by two cognitive processes acting in concert: decoding and reasoning (Sabbagh, 2004). The former refers to the ability to decode others' mental states on the basis of observable information. This process has historically been studied using the Baron‐Cohen Eyes‐test (Adams et al., 2010; Baron‐Cohen et al., 1999; Castelli et al., 2010; Gunther Moor et al., 2012), which requires one to infer mental states of others on the basis of their eyes' expression. By contrast, reasoning corresponds to processes used to infer others' emotional states from social situations and predict their emotional reactions. The neural correlates of reasoning have mainly been explored through emotional inference tasks, based on verbal social situations (Corradi‐Dell'acqua et al., 2014; Hynes, Baird, & Grafton, 2006) or cartoons (Atique, Erb, Gharabaghi, Grodd, & Anders, 2011; Schnell, Bluschke, Konradt, & Walter, 2011; Sebastian et al., 2012; Völlm et al., 2006). Despite the growing literature on the neural substrate of affective ToM (for meta‐analysis, see Molenberghs, Johnson, Henry, & Mattingley, 2016), no study has specifically assessed whether the complexity of the emotional state inferred from others can influence the brain regions sustaining either the decoding of or reasoning on affective mental states. Indeed, as far as we know, investigations using Baron‐Cohen Eyes‐test did not contrast the inference of others' basic (e.g., joy, sadness, anger, surprise, fear, and disgust) versus complex mental states, and the few reasoning studies comparing complex to basic (Burnett, Bird, Moll, Frith, & Blakemore, 2009; Gilead, Katzir, Eyal, & Liberman, 2016; Sturm et al., 2013) or neutral emotions (Roth, Kaffenberger, Herwig, & Brühl, 2014) involved self‐referential processes rather than ToM per se.

In this context, self‐conscious emotions (SCE) such as shame, guilt, embarrassment, and pride are of particular interest as they differ in several ways from basic emotions (BEs). These emotions involve inferences about other people's evaluations of the individual (Leary, 2007). First, although existing across different cultures (Cordaro et al., 2017), SCE are less strongly associated with universal facial expressions than BE (Zinck, 2008). Therefore, identification of SCE relies on distinct expression sets, including body posture and head movement combined with facial expressions (Keltner, 1995; Tracy & Matsumoto, 2008). Second, individual's SCE are highly evolved and emerge later in development than BE (Zinck, 2008) as they need both self‐awareness and self‐representations (Tracy & Robins, 2007), that is, capacities to evaluate one's self. In addition, SCE require to infer mental states from others (Beer, Heerey, Keltner, Scabini, & Knight, 2003). Thus, it is not surprising that the development of SCE experiences, and knowledge relative to these emotions, occur in conjunction with the acquisition of the ToM (Lagattuta & Thompson, 2007). Finally, compared to BE, SCE are more complex and involve elaborated cognitive processes that integrate and evaluate behaviors according to rules, expectations, and goals (Tracy & Robins, 2004b, 2007). These genuine differences might explain why SCE are sustained by specific brain regions such as the medial prefrontal and anterior cingulate cortices (Jankowski & Takahashi, 2014), but also the anterior insula (Sturm et al., 2013). Thus, SCE are not just more complex than BE, they are inherently different by nature and are more difficult to identify. The purpose of this study wasn't to evaluate the personal experience of these emotions per se. Instead, we wanted to evaluate the differences in the treatment of these emotions through the lens of the affective ToM.

In the present study, we assessed for the first time if the nature of the emotion can influence the cerebral substrate of affective ToM. Specifically, we used an affective ToM task involving sequentially reasoning and decoding processes to assess if the inference of others' SCE versus BE relies on different neural substrates. Unlike most previous studies that assessed affective ToM through thumbnails and verbal scenarios (e.g., Atique et al., 2011; Hynes et al., 2006), we used an ecological functional MRI (fMRI) paradigm with original movies to provide emotional situations and expressions that individuals encounter in the social world. The validation of this task as an ecological tool to assess the affective ToM constitutes one of the strengths of this study for the characterization of ToM processes. Indeed, as many studies have shown (for example, see Chen et al., 2017; Lecce, Ceccato, & Cavallini, 2018), the use of ecological tasks for the assessment of ToM is crucial to (a) propose conditions closer to the complexity of social interactions in daily life and thus (b) obtain results that better reflect complex ToM processing in reality. In addition, this study proposes to shed light on the effect of emotional complexity of different affective ToM processes, which until now had not been clearly elucidated in the literature. As SCE are inherently more complex than BE, we hypothesized that their inference should be associated with more activation of the affective ToM network, regardless of the process involved. Furthermore, due to their lack of universal facial expression, we expected that the decoding of others' SCE relies more on body clues than the decoding of others' BE. We therefore hypothesized a differential involvement of the brain regions involved in body, but not face, processing for the decoding of these two types of emotions.

## 2 MATERIALS AND METHODS

### 2.1 Participants

Participants were recruited from different sources such as advertisements in electronic media and advertisements posted in the community. For each subject, a telephone pre‐screening interview was first conducted in order to collect general information, ensure the absence of contraindications for MRI and the fit with certain inclusion criteria (i.e., be right‐handed, native French speakers, and between 20 and 30 years old). All participants then underwent a medical examination with a physician to ensure the absence of abnormality on standard clinical examination, neurological or psychiatric disorder, and history of addiction. Specifically, subjects were excluded if they had a psychiatric (e.g., depression) or neurological (e.g., stroke) medical history, if they were taking antidepressants, sleeping pills, and/or anxiolytics, if they had an endocrine or liver disease, a history of cancer (in the past 5 years), if they were epileptic, claustrophobic, pregnant, or if they consumed more than two or three alcoholic drinks (depending on woman or man) per day. Thus, 21 healthy young subjects (11 women and 10 men, mean age = 23.9 ± 2.4 years, mean years of education = 13.6 ± 1.7 years) were recruited and underwent an fMRI scan.

The regional ethics committee (CPP NordOuest III) approved the study and all the participants gave their written informed consent prior to participation. All procedures performed were in accordance with the ethical standards of the institutional and/or national research committee and with the 1964 Helsinki declaration and its later amendments or comparable ethical standards.

## 3 EXPERIMENTAL DESIGN

### 3.1 Pierre and Marie fMRI task

**Figure 1:**
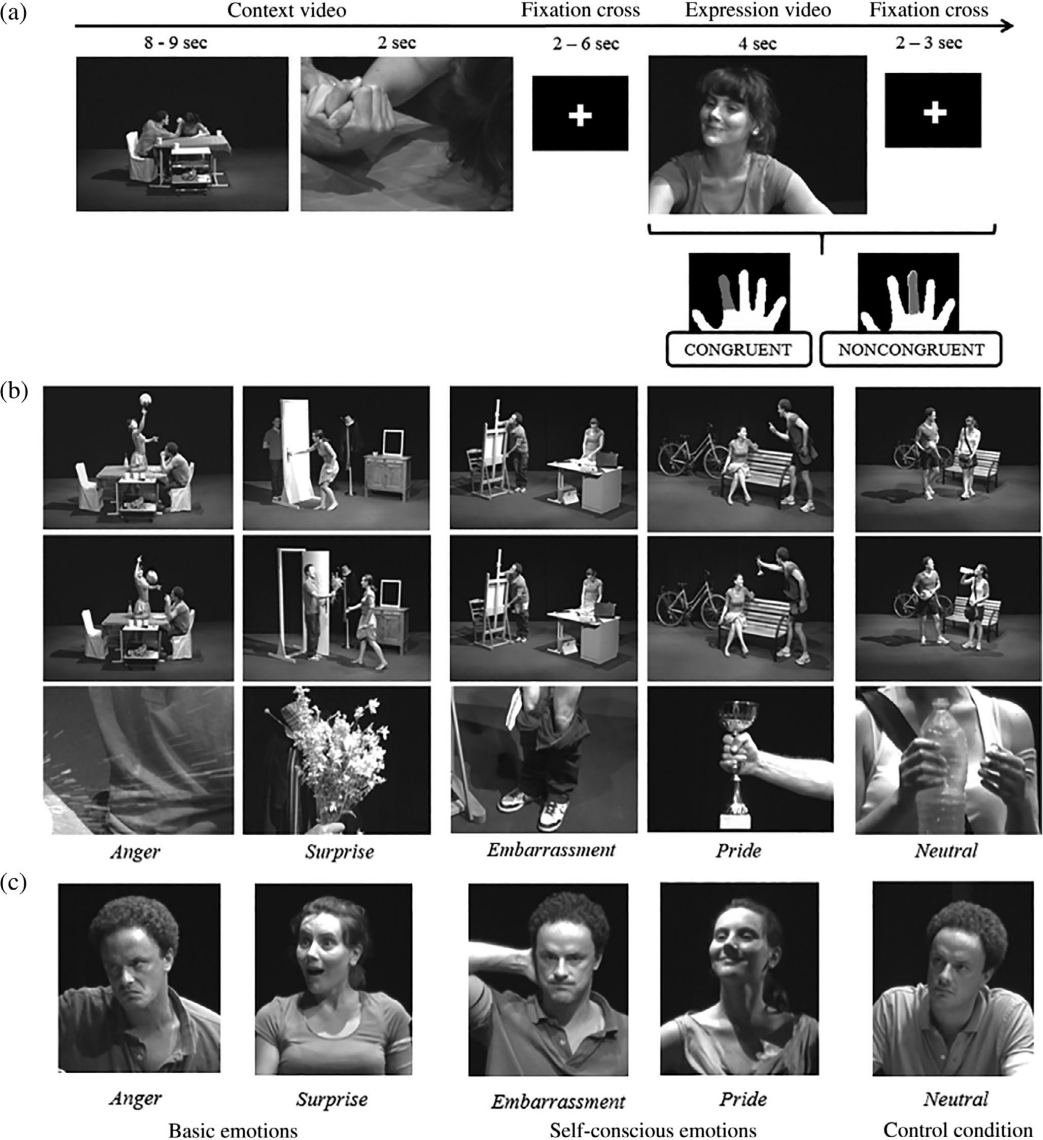
Design of the fMRI task (a) and examples of context (b) and expression (c) videos for the five emotions. *Note*: A pink armband is used during the context videos to designate the actor that will then express an emotion. In this case, Pierre is wearing the armband for anger, embarrassment, and pride, and Marie for surprise and neutral (b)

SCE and BE inferences were assessed through an ecological task, using black and white mute videos featuring two characters (Pierre and Marie). They were introduced as roommates living together for a short time (see Figure 1a for an overview of the experimental design). The scenes occurred in five different places (dining and living rooms, office, entrance, and park). We intentionally limited the number of distracting elements from the background scene to maintain participant's attention focused on the two protagonists. Videos were divided into two parts. First, the “context videos” (reasoning phase) showed the two characters interacting in a social situation (see Figure 1b for examples of Context videos). A full shot (8–9 s) composed this part, giving an overview of the context, followed by a close‐up shot (2 s) providing a deeper understanding of the situation. This situation was followed by a fixation cross (semi‐random duration of 2–6 s) and then by the “expression videos” (decoding phase) in which one of the two protagonists had an emotional reaction in response to the situation (4 s—see Figure 1c for examples of expressions).

Context videos were designed to elicit an emotion in one of the two characters, designated by a pink armband. Five distinct expressions, grouped under three conditions, could thus be conveyed by the stories: embarrassment or pride (SCE), anger or surprise (BE), or neutral (i.e., absence of emotion). This latter situation was used as a control condition and refers to situations that did not elicit any emotional feeling to the protagonist. The choice of the four emotions was motivated by conceptual and pragmatic reasons. Specifically, we aimed at controlling for the emotional valence by including both negative (embarrassment and anger) and positive (pride and surprise) emotions. The choice of these emotions was further driven by (a) the ability to differentiate one emotion from another (e.g., joy and pride would have been conceptually too close to be properly distinguished), (b) the control of the participant's emotional reaction associated with the movies (e.g., disgust stories are likely to lead to disgust reaction in the subject watching the movie), and (c) the feasibility of inventing distinct scenarios that could be understood in <10–11 s through a movie with no sound.

Expression videos consisted of a medium close‐up shot, including both the upper part of the body and the face of the actor designated by the pink armband. This actor expressed one of the five expressions (i.e., embarrassment, pride, anger, surprise, or neutral) either in adequacy or not with the situation. Half of the expressions was congruent with the situation, and the other half was noncongruent and consisted equitably of the other expressions (e.g., for the 24 context of pride, 12 expressions conveyed pride, 3 embarrassment, 3 anger, 3 surprise, and 3 neutral).

The experimental material has been validated through different steps, with pretests on a total of 191 healthy subjects, different from those recruited in this study (see Supplementary material for additional details on the Validation procedure of the material). First, 44 healthy subjects rated the degree of emotion elicited by 210 invented verbal stories, describing everyday life social situations. The most relevant situations (*n* = 152) were then chosen to be filmed. Once edited, the videos were submitted to new pretests. Specifically, 125 healthy subjects were asked to assess if the context and/or the expression videos properly conveyed the expected emotions when presented separately. Thus, we made sure that subjects made a correct inference for our stories. This therefore provided a measure of validity of the emotion expressed in each video. In addition, 22 different subjects rated the congruency of the expressions with their situations when both context and expression videos were presented together. Based on these pretests, we selected the best 120 videos to obtain an equal distribution across both emotion and congruency conditions (for details, see Table S1, and for examples of selected context and expression situations, see online supplementary material Videos). The male actor (*Pierre)* was the target of the inference (i.e., wearing the pink armband) in 59 videos versus 61 for the female actor (*Marie*).

The experimental task was divided into 4 fMRI runs of about 11 min each. During each run, 30 videos (context followed by their expression part) were broadcasted with an inter‐stimuli interval of 2–3 s between each video. Subjects were instructed to focus their attention on the protagonist wearing the pink armband and to think to what he/she might feel during the context videos (reasoning process). They were then asked to judge, as fast as possible, whether the emotional reaction presented during the expression videos was congruent or not with the situation (decoding process). The subjects do not have to properly label the emotions but simply have to make a judgment of congruency between the context and the expression videos, implying only two possible answers. This experimental choice was notably motivated to make the task accessible to other types of population (aging and neurodegenerative disease) and to propose an implicit evaluation of these ToM components. All subjects were trained prior to the scan with videos that were not used for the fMRI task.

### 3.2 Localizer

In order to perform complementary analyses in the brain regions specifically devoted to face and body processing, all participants underwent a standard 1‐back localizer task for about 9 min, after the Pierre and Marie task. The task included four different conditions (Face, Body, Chair, and Rest). The first three were presented in blocks of 13.5 s, during which 9 photographs (including 2 similar) were shown successively (1 second of presentation; inter stimuli interval = 0.5 s). The block order (Face, Body, and Chair) was semirandom and was followed by 12 s of rest, during which the subjects had no particular instructions. Body and chair photographs were similar than the one used by Peelen and Downing (2005), and face photographs were selected from the “The Glasgow Face Matching Test” (Burton, White, & McNeill, 2010).

## 4 MRI DATA ACQUISITION

Two scanning sessions were performed with a 3 Tesla Philips Achieva Scanner (Caen, Cyceron). In the first acquisition session, a high‐resolution T1‐weighted anatomical image was first acquired using a 3D fast field echo sequence (3D‐T1‐FFE sagittal, repetition time (TR) = 20 ms; echo time (TE) = 4.6 ms; flip angle 10°; 180 slices; no gap; slice thickness = 1 mm; field of view (FOV) = 256 × 256 mm^2^; acquisition voxel size = 1 × 1 × 1 mm^3^), followed by a high‐resolution T2‐weighted anatomical image (2D‐T2‐SE sagittal, SENSE factor = 2; TR = 5,500 ms; TE = 80 ms; flip angle = 90°; 81 slices; no gap; slice thickness = 2 mm; FOV = 256 × 256 mm^2^; acquisition voxel size = 2 × 1 × 1 mm^3^) and a non‐EPI T2 star image (2D‐T2 star‐FFE axial, SENSE factor = 2; TR = 3,505 ms; TE = 30 ms; flip angle = 90°; 70 slices; no gap; slice thickness = 2 mm; FOV = 256 × 256 mm^2^; matrix = 128 × 128; acquisition voxel size = 2 × 2 × 2 mm^3^). In the second acquisition session, an EPI T2 star image, similar to that of the first session, and five functional runs (four for the Pierre and Marie task and one for the localizer task) were acquired. The six initial volumes of each run were discarded to control for magnetic saturation effects. Functional data were then acquired using an interleaved 2D T2 star EPI sequence designed to reduce geometrical distortions and magnetic susceptibility artifacts (2D‐T2 star‐FFE‐EPI axial, SENSE factor = 2; TR = 2,600 ms; TE = 30 ms; flip angle = 80°; 42 slices; no gap; slice thickness = 2.8 mm; FOV = 224 × 224 mm^2^; acquisition voxel size = 2.8 × 2.8 × 3.0 mm^3^; 275 volumes per run for the Pierre and Marie task and 231 for the localizer task).

## 5 BEHAVIORAL DATA ANALYSIS

The behavioral data of the fMRI task were analyzed using Statistica 10.0 (StatSoft, Tulsa, OK). Analyses of variance (ANOVAs), followed by Bonferroni's posthoc analyses, were conducted to compare emotional conditions (SCE, BE, and neutral) for both the percentage of correct answers and the reaction time for correct answers.

## 6 FMRI DATA PREPROCESSING AND ANALYSIS

Statistical analyses of functional volumes were performed in a two‐step analysis using the Statistical Parametric Mapping 8 software (SPM8, Wellcome Department of Imaging Neuroscience, Institute of Neurology, London, UK).

Preprocessing was conducted according to a procedure developed in our laboratory (Villain et al., 2010). Briefly, after slice timing correction, data were realigned with the first volume of each run. Geometric EPI distortions were then corrected as follows: the mean EPI image was coregistered onto the non‐EPI T2 star volume of the functional session, the non‐EPI T2 star volume of the functional session was coregistered onto the anatomical one, the non‐EPI T2 star volume of the anatomical session was then coregistered onto the T2 image, and finally, the T2 volume was coregistered onto the T1 image. After coregistration, functional images were normalized using the parameters derived from the nonlinear normalization of individual gray‐matter T1 images to the template of the Montreal Neurological Institute (MNI). Finally, normalized images were smoothed using an 8‐mm FWHM kernel.

For each participant, we first performed a general linear model including both context and expression videos from the Pierre and Marie task. More precisely, context videos (from their initial presentation until the fixation cross) were modeled according to the emotion condition (SCE, BE or neutral). For expression videos, stimuli associated with a correct response were modeled depending on both emotion type (SCE, BE or neutral) and congruency with the situation (congruent or noncongruent). Expression videos associated with an incorrect or lack of response were included as a variable of noninterest. Further regressors of no interest were the six realignment parameters to account for linear residual motion artifacts. For each participant, the main effects of SCE, BE, and Neutral conditions were computed separately for context and expression videos.

Second‐level analyses consisted in comparisons between the types of emotion in each process involved. Thus, two distinct flexible factorial models were performed to assess the effect of emotion type during reasoning (context videos) and decoding (expression videos) processes. To do so, we contrasted each type of emotion to the two others (i.e., SCE versus Neutral, SCE versus BE, BE versus Neutral). For all analyses, comparisons were limited to a gray matter mask including voxels where the mean gray matter probability of the group was superior to 0.3.

The family‐wise error (FWE) corrected threshold was set at *p* < .05, and the cluster extent at *k >* 100 mm^3^ (i.e., *k* > 13 voxels). Note that all significant clusters survived to the corrected FWE‐threshold *p* < .05 at cluster level.

Finally, to further understand the difference between the two emotional conditions, we performed analyses in the brain regions specific to face and body processing, that is, the fusiform face area (FFA) and the extrastriate body area (EBA). To do so, we computed for each participant a general linear model for the localizer task, regressing out each experimental condition (faces, bodies, chairs, and rest) and the motion parameters. We then used the preprocessed images in native space (i.e., smoothed using a 6‐mm FWHM kernel but not normalized) to identify both the FFA and EBA of each subject using the contrasts *Faces > Chairs* and *Body > Chairs*, respectively. In order to preserve a better spatial accuracy, the smooth is different in native space, compared to normalized images. Regions of interest (ROIs) were defined using an in‐house Matlab script based on the ROI‐extraction toolbox (https://github.com/iancharest/roi-extraction). In each participant, individual peak maxima in bilateral FFA and EBA were identified using their corresponding contrast. From each peak, the ROI was then built by including sequentially the most significant contiguous voxels starting from the chosen voxel and up to *n* voxels (*n* = 50 in the present study). Using this procedure, we were able to create for each subject left and right ROIs of similar cluster sizes (*k* = 50 voxels) for both FFA and EBA. An illustration of these ROIs is provided in online supplementary material Figure 1.

We then used these ROIs to extract the mean signal during the decoding process for the two emotional conditions (i.e., SCE and BE). Note that left and right ROIs were pooled together to extract the mean signal. Using the ROI type as one factor (FFA and EBA) and emotion complexity as a second factor (SCE and BE), 2 × 2 repeated measures ANOVA were carried out on the mean signal of each region, followed by Bonferroni's posthoc analyses.

## 7 RESULTS

### 7.1 Behavioral results

Participant performances ranged between 78 and 97% (90.9 ± 4.3%) of correct answers on the Pierre and Marie task. Between‐condition comparisons showed that performances were significantly superior for SCE (*p* < .001) and BE (*p* < .001) compared to the neutral condition. No significant difference was noted between the two emotional conditions (*p* = .58). Similarly, we found an effect of the type of emotion on reaction times, with judgments on neutral expressions being significantly slower than those on SCE (*p* < .001) and BE (*p* < .001) expressions. No significant difference was observed between the two emotional conditions (*p* = .99).

### 7.2 Imaging results

#### 7.2.1 Reasoning process

**Figure 2:**
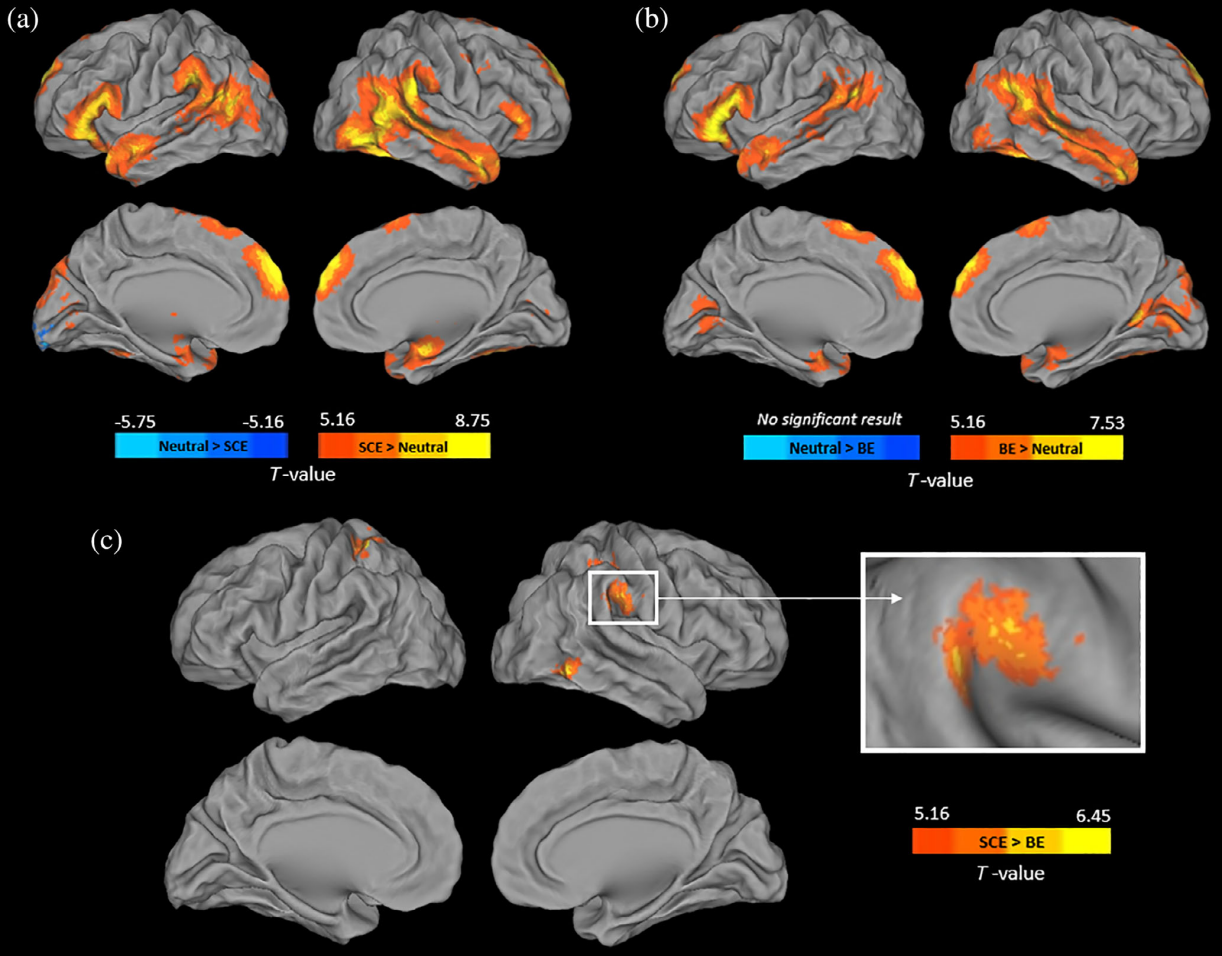
Voxel‐wise analyses showing differences of activation during the reasoning process between the neutral and SCE (a), neutral and BE (b), and SCE and BE (c) conditions (FWE‐corrected *p* < .05 and cluster extent *k* > 100 mm^3^). *Note*: Results are shown in neurologic convention. BE, basic emotions; SCE, self‐conscious emotions

During Context videos, *SCE* were associated with greater activation than the *Neutral condition* in bilateral prefrontal dorsomedial, orbitofrontal, inferior frontal, and superior temporal regions (right superior temporal sulcus), extending into the inferior occipital cortex, fusiform and supramarginal gyri, as well as in the thalamus and amygdala (Figure 2a, see Table S2 for peak details). The contrast *BE > Neutral* revealed a similar pattern of differences, although less spatially extended (Figure 2b). The contrast *Neutral > SCE* only showed a difference of activation in the left lingual gyrus and no brain region was more activated for the *Neutral condition* than *BE*.

Comparisons between the two emotional conditions showed greater activations for *SCE* than *BE* in the right temporoparietal junction, right inferior temporal region, and bilateral superior parietal lobules (Figure 2c). The opposite contrast (i.e., *BE* > *SCE)* did not reveal any significant result.

#### 7.2.2 Decoding process

**Figure 3:**
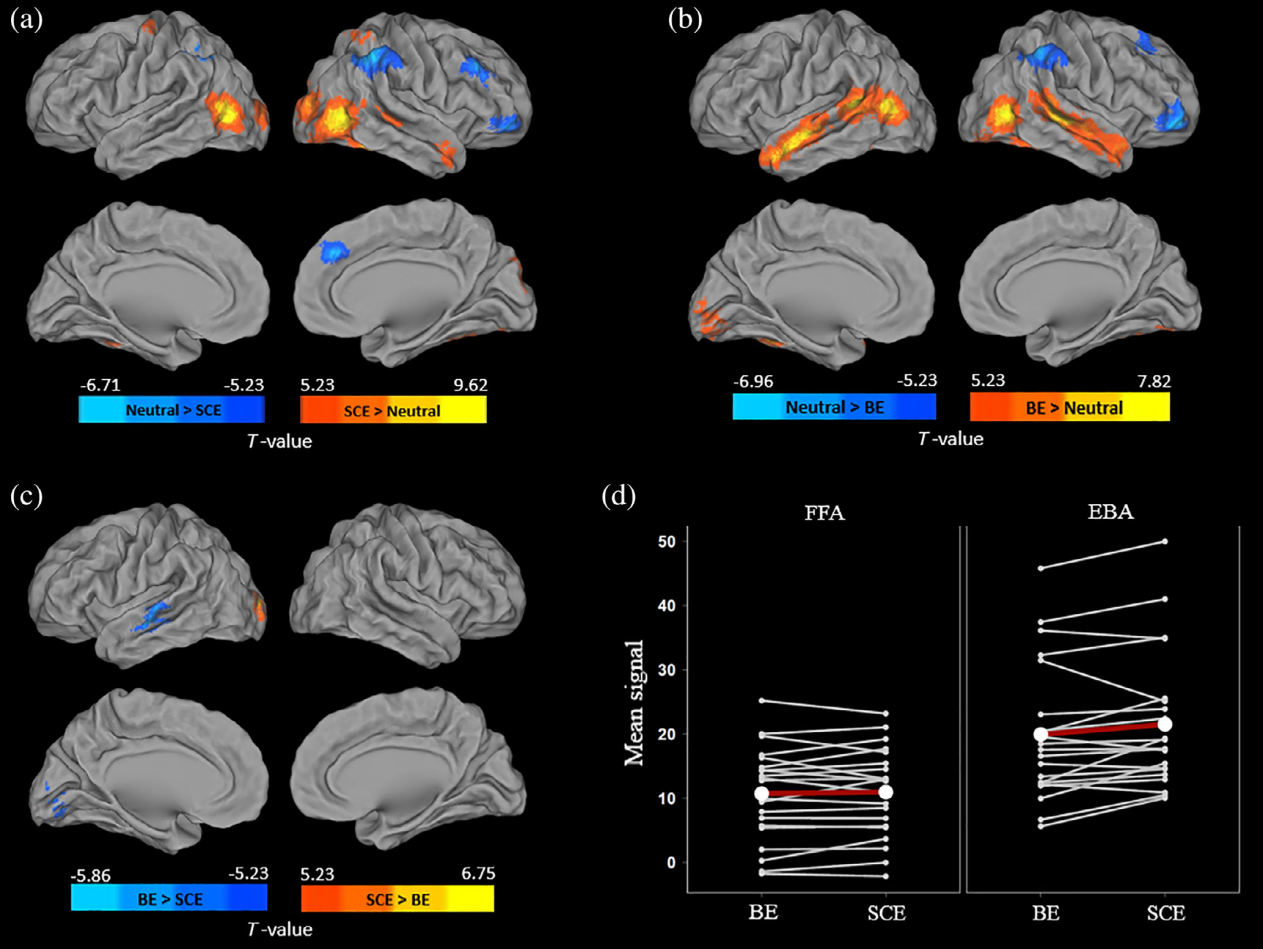
Voxel‐wise and ROI analyses showing differences of activation during the decoding process between neutral and SCE (a), neutral and BE (b), and SCE and BE (c,d) conditions (FWE‐corrected *p* < .05 and cluster extent *k* > 100 mm^3^). The plot (d) represents the mean signal for the two emotional conditions in the FFA and EBA for each individual (white line) and at the group level (red line). *Note*: Results are shown in neurologic convention. BE, basic emotions; EBA, extrastriate body area; FFA, fusiform face area; SCE, self‐conscious emotions

During the expression videos, the *SCE* condition was associated with greater activation than the neutral condition in the bilateral inferior occipital regions, right temporal pole, and superior temporal sulcus, left fusiform, and precentral gyri (Figure 3a, see Table S3 for peak details). Conversely, the right middle frontal gyrus, right orbitofrontal, and bilateral inferior parietal regions showed more activation for *Neutral* than *SCE* conditions.

Compared to the *Neutral condition*, *BE* presented with greater activations in bilateral temporo‐occipital regions, middle, superior temporal, and fusiform gyri. The reverse contrast (i.e., *Neutral > BE*) revealed higher activation in the right superior and middle frontal gyri, and right inferior parietal regions (see Figure 3b).

Comparison between the two emotional conditions showed that *SCE* was associated with more activation than *BE* in the left occipital middle regions. By contrast, the left inferior temporal regions and the left lingual gyrus showed more activation during *BE* than *SCE* (see Figure 3c).

Analyses focused on the face and body ROIs, obtained with the localizer task, revealed a significant main effect for the ROI (F[1, 20] = 28.52, *p* < .001) but not for the emotion (F[1, 20] = 3.73, *p* = .07). The interaction between ROI and emotion was significant (F[1, 20] = 4.83, *p* < .05). Posthoc analysis revealed that the EBA (*p* = .009), but not the FFA (*p* = .99), was significantly more activated for SCE than BE (Figure 3d).

## 8 DISCUSSION

In spite of a better characterization of ToM neural substrates since two decades, it is still unclear whether the nature of the emotional state inferred from others can influence the pattern of brain activations associated with affective ToM. In the present study, we used an ecological task to compare brain activations during the decoding of and reasoning on others' SCE, BE, and neutral mental state. Our results showed that compared to neutral states, the inference of self‐conscious and basic emotional states from others requires more activation in a distributed network including several key regions of affective ToM. Direct comparisons between emotional conditions revealed more activation for SCE than BE in the right temporoparietal junction, inferior temporal and superior parietal regions during the reasoning process, and in the left middle occipital regions during the decoding process. These results provide further support to a distinct neural recruitment for SCE and BE processing and demonstrate that the complexity of the emotion inferred to others can influence the recruitment of the ToM network.

Brain regions recruited during reasoning (i.e., context videos) on SCE and BE encompass several brain regions involved in affective ToM (Abu‐Akel & Shamay‐Tsoory, 2011) such as the dorsomedial, orbitofrontal and inferior prefrontal regions, temporoparietal junction, superior temporal sulci, temporal poles, and amygdala. These regions play a key role in the inference of mental states and the treatment of social and emotional information (Mitchell, 2009). Interestingly, our results showed that some of these regions are also elicited when the inference of others' SCE and BE is based on facial/body expressions. Indeed, we found a higher involvement of the temporal pole, superior temporal, and fusiform gyrus for the decoding of emotional versus neutral states. The recruitment of these regions may therefore be necessary for the inference of an emotional state from other individuals, independently of the specific process itself. The temporal pole is considered as a core region for the management and storage of social semantic concepts (Olson, Plotzker, & Ezzyat, 2007; Zahn et al., 2009). Previous studies reported its involvement in the recognition of visual cues and in both emotional processing and mentalizing (Olson et al., 2007). This region is thought to provide a framework, which associate recognized emotions with emotional scripts of affective ToM based on semantic knowledge (Mier et al., 2010). By contrast, the superior temporal gyrus would contribute to ToM processing by decoding social actions or intentions (Schultz et al., 2003). This region is also involved in motion perception and more generally in the identification and the representation of complex goal‐directed motions (Mier et al., 2010). Although we did not directly compare the activations during the decoding of and reasoning on others' emotional states, it is interesting to note the specific involvement of the dorsomedial prefrontal cortex during the reasoning process. This is in line with a previous study showing that inferring a plausible social cause from a video (reasoning) is associated with greater activation in the dorsomedial prefrontal cortex than the identification of emotionally relevant motor behavior (Spunt & Lieberman, 2012). It is thus possible that this region plays a specific role in the manipulation of and reasoning on mental states (Van Overwalle, 2011), and/or in the processing of social interactions involving to reason on complex natural stimuli (such as movies in our fMRI task) (Wagner, Kelley, Haxby, & Heatherton, 2016).

The comparison between the two emotional conditions showed that the inference of others' SCE from social situations (i.e., context videos) requires more activation than BE in temporal and parietal regions. Specifically, we found differences in the right temporoparietal junction, a region that has been previously involved in SCE processing (Berthoz, 2002; Burnett et al., 2009; Burnett & Blakemore, 2009). This structure plays a critical role in ToM (Van Overwalle, 2009) and has been proposed to be selective for the attribution of mental states to others (Saxe & Powell, 2006; Saxe, Schulz, & Jiang, 2006). Interestingly, a previous study has shown that activation in the right temporoparietal junction increases with the level of representational complexity of the mental state inferred (Abraham, Werning, Rakoczy, von Cramon, & Schubotz, 2008). This is consistent with the idea that SCE requires a higher level of inference than BE (Burnett & Blakemore, 2009). Indeed, to be experienced, SCE requires insight into the mental states of others whether they are physically present, imagined, or represented by the concept in societal norms (Burnett et al., 2009). Hence, their inference to someone requires not only to adopt its own perspective, but also to consider other peoples' perspectives and make inferences on their emotions (Olsson & Ochsner, 2008). We therefore hypothesize that the increased activation in the right temporoparietal junction may reflect the simultaneous consideration of both protagonist mental states, necessary to properly attribute SCE to one of them. It is worth mentioning that growing evidence suggests distinct functional role of the right temporoparietal junction according to the antero‐posterior axis (Mars et al., 2012; Schurz, Radua, Aichhorn, Richlan, & Perner, 2014). While the right posterior temporoparietal junction would be exclusively involved in social cognition (i.e., consistently recruited by ToM tasks such as false belief task), the right anterior temporoparietal junction is associated with both attentional (reorienting of attention) and ToM processes (for meta‐analysis, see Krall et al., 2015). According to Krall and collaborators (2015), the overlap of reorienting of attention and false belief in the right anterior temporoparietal junction may reflect the fundamental requirement of shifting between mental states to perform ToM tasks. Given that our result predominated in the anterior part of the temporoparietal junction, it is possible that it results from the greater attentional requirement (i.e., more shifting between both protagonist mental states) for SCE than BE inferences.

During the decoding process, emotion comparisons showed that the decoding of others' SCE was associated with increased left medial occipital activation compared to BE. Since there is no discrete and universal facial expression associated with SCE (Lewis, 1997), this difference may reflect the fact that SCE necessitate the observation of bodily action or postural changes more than facial clues, hence requiring a finer decoding of these signals (Tracy & Robins, 2004a). In agreement with this idea, we found that the EBA, but not the FFA, was more activated during the decoding of SCE versus BE. This emphasizes the importance of body features to recognize and infer SCE to others. Previous neuropsychological studies have shown that SCE decoding requires more complex patterns than BE due to the importance of differentiated gestures, body, and head postures (Zinck, 2008).

The lack of functional differences in ToM‐related regions during the decoding process contrasts with the assumption in the literature stipulating that the inference of complex emotions from facial/body clues implies more sophisticated cognitive processes than BE (Tracy, Robins, Schriber, & Solomon, 2011). This hypothesis is based on the fact that these emotions are (a) acquired and (b) recognized later in the development (Lagattuta & Thompson, 2007), and that (c) their recognition can be specifically impaired (Sturm, 2006). However, to our knowledge, no study has directly compared the functional differences between the decoding of others' complex versus BEs. Using an ecological fMRI task, we failed to report significant differences in ToM regions between these two types of emotions. Given the specific design of our task, it is possible that emotional differences in ToM requirement were diminished due to the dynamic nature of our stimuli, which facilitated their processing. In addition, subjects had to perform a congruency judgment rather than to label the emotional expressions, which may also affect the ToM requirement. Further studies are thus needed to provide a deeper understanding of the experimental conditions altering the recruitment of ToM network during decoding processes.

This study has some limitations. First, the neutral condition leads to lower performances and longer reaction times than the emotional conditions. Furthermore, higher activations in frontoparietal regions were associated with this condition during the expression videos. These regions cover the dorsal attentional network, which play a role in the selection of stimuli according to internal objectives or expectations (Corbetta, Patel, & Shulman, 2008). Taken together, these results may reflect the greatest attentional cost of the neutral condition, possibly due to the unusual nature of neutral expressions (i.e., inexpressive expressions during 4 s), and/or the higher control resulting from greater uncertainty in the response. However, this limitation only concerned the expression videos. Second, despite the plausible influence of the context on the decoding process (e.g., Carroll & Russell, 1996), we pooled together congruent and noncongruent expressions in our statistical model. This experimental choice was done to limit the effect of the context and to assess the decoding process involved in both cases. In this regard, it is worth mentioning that congruent and noncongruent expressions were similarly counterbalanced across emotional conditions and that we did not find an interaction between congruency and emotion type for the performance or reaction times (data not shown). Also, in the present study, we focused on a limited set of basic and complex emotions to assess the influence of emotional complexity on ToM‐related brain activations. Future studies could use distinct BE or SCE to further assess the generalization of our results. Finally, correct answers were defined based on the congruency judgment, and we cannot entirely rule out that, for some items, participants did not properly recognize the emotions associated with the reasoning and/or decoding phases. Yet, it is worth mentioning that our pretests indicated that 85.7 and 87.9% of the emotions were correctly recognized for the reasoning and decoding phases, respectively. It is therefore likely that a correct congruency response was associated with correct recognition of the emotions associated with the reasoning and decoding phases.

## 9 CONCLUSIONS

Overall, using an ecological design, our study highlights an extended pattern of common activations for affective ToM, regardless of the emotion or process. Furthermore, our results demonstrate differences in activation patterns according to the complexity of the emotions. Specifically, we reported that, compared to BE, the inference of others' SCE from a social situation is associated with greater activation in the right temporoparietal junction. This result further supports that SCE requires a higher level of inference and emphasizes that the right temporoparietal junction may be sensitive to emotional complexity. Altogether, our results reinforce the interest in dissociating SCE from BE and studying them distinctly and through ecological paradigms in future studies. This will be especially true in the context of pathological conditions in which deficits of ToM are hard to detect and characterize with classic tools.

## CONFLICT OF INTEREST

Authors declare that they have no conflict of interest.

## Supporting information


**Appendix S1**: Supporting Information

**Appendix S2**: Context Videos

**Appendix S3**: Expression Videos

## Data Availability

The data that support the findings of this study are available from the corresponding author upon reasonable request.
