# Supplementary material for: Influence of emotional complexity on the neural substrates of affective theory of mind
Source: Hum Brain Mapp. 2019 Sep 30;41(1):139–49. doi: 10.1002/hbm.24794 (PMC7267895; doi:10.1002/hbm.24794)
Supplement: Supplementary file 1 — Appendix S1: Supporting Information [file HBM-41-139-s001.doc]

**Supplementary Material**

# **Legends**

**Task Validation Procedure.** Description of all the pretests

**Table 1.** Repartition of the different emotions in the Decoding and Reasoning phases. Congruent emotions (same emotion for Decoding and Reasoning phases) are shown in pale orange.

**Figure 1.** Example of bilateral fusiform face area (a) and extrastriate body area (b) in some subjects, obtained in native space.

**Table 2.** Voxel-wise results showing the difference between self-conscious, basic, and neutral conditions during Context videos (FWE-corrected p <0.05 and cluster extent k >100 mm^3^).

**Table 3.** Voxel-wise results showing the difference between self-conscious, basic, and neutral conditions during Expression videos (FWE-corrected p <0.05 and cluster extent k >100 mm^3^).

**Videos.** Example of videos of Pierre and Marie Task (Context followed by Expression).

| **Name file** | **Emotion associated with Context** | **Emotion associated with Expression** | **Congruence** |
| --- | --- | --- | --- |
| Context_01_Embarras_Congruent.avi | Embarras (Self-conscious) |  | Yes |
| Expression_01_Embarras_Congruent.avi |  | Embarras |
| Context_02_Pride_Non_Congruent.avi | Pride (Self-conscious) |  | No |
| Expression_02_Anger_Non_Congruent.avi |  | Anger |
| Context_03_Anger_Congruent.avi | Anger (Basic) |  | Yes |
| Expression_03_Anger_Congruent.avi |  | Anger |
| Context_04_Surprise_Non_Congruent.avi | Surprise (Basic) |  | No |
| Expression_04_Embarras_Non_Congruent.avi |  | Embarras |
| Context_05_Neutral_Congruent.avi | Neutral |  | Yes |
| Expression_05_Neutral_Congruent.avi |  | Neutral |
| Context_06_Neutral_Non_Congruent.avi | Neutral |  | No |
| Expression_06_Neutral_Non_Congruent.avi |  | Surprise |

**Task Validation Procedure.** Description of all the pretests

As stated in the manuscript, the experimental material has been validated through different steps, with pretests performed on a total of 191 healthy subjects, distinct from those included in the study.

First of all, in order to select the stories to be filmed, we invented 210 scenarios that elicit pride, surprise, embarrassment, anger or no emotion (neutral). To assess if these stories properly elicited the expected emotions, we conducted written pretests on 44 healthy subjects (mean age = 42.5 ± 23.3 years). Specifically, participants were asked to judge the emotional feelings of a character in a given situation. For each story, subjects rated the degree (on a scale from 0 to 10) of pride, surprise, embarrassment and anger elicited by the situation, or had to specify if the story was not eliciting any emotion (neutral). Subjects also performed a judgment of familiarity on the stories. The best stories (n=152) were then chosen to be filmed.

Once edited, the videos were submitted to new pretests to ensure that they properly convey the expected emotions. A total of 147 subjects contributed to this second phase, throughout which three distinct pretests, with distinct objectives, were performed.

1) To reach a large sample of subjects, we developed a website to watch the videos and answer questions about the emotions elicited/expressed. The website link was sent to researchers’ relatives and diffused through the mailing list of the University of Caen. The data of 86 subjects (mean age = 41 ± 25.5 years) were sufficiently complete to be analyzed. In this pretest, subjects watched the videos of Context and Expression separately and had to rate, after each video, the familiarity of the scene, the ease of understanding, and the emotions expressed. The objective here was to assess the relative specificity of each emotion, i.e., how much one emotion was strongly associated to a stimulus or if there was a combination of several emotions (see Table below, % correct emotion > others).

2) The objective of the second pretest was to assess, separately for the videos of Context and Expression, which emotion was the most elicited/expressed by the videos. This pretest was performed by a Master student in Psychology on 39 healthy subjects (mean age = 44 ± 22.6 years). Following each video of Context, subjects had to choose which emotion will be felt by the protagonist wearing the pink armband. Similarly, after each video of Expression, subjects had to choose which emotion was expressed by that same character (see Table below, % correct answers). To answer, subjects could choose among the 5 possible answers (pride, surprise, embarrassment, anger or no emotion) of the experiment.

3) The purpose of the third pretest was to assess the congruency between the Context and Expression videos. In other words, we aimed here to ensure that a Context of “pride”, followed by an Expression of “pride”, is correctly labeled as congruent. This pretest was performed by a Master student in Psychology on 22 subjects (mean age = 35.5 ± 17.7 years). Subjects watched the video of Context, followed by the video of Expression, and were then asked to perform the congruency judgment as fast as possible (see Table below, % correct congruency answers). This task was therefore very close to the one used under fMRI. Following the judgment of congruency, subjects also rated how much the emotion was adapted (or non-adapted depending on the answer) to the situation, what emotion was expressed by the character (according to the 5 possibilities), what was the intensity of the emotion expressed by the character, and how much the story was easy to understand.

Table. Percentage of correct recognition of our three last pretests for each emotion, during the Reasoning and Decoding phases and for the congruency between both phases.

|  | Reasoning phase | | Decoding phase | | Reasoning + Decoding |
| --- | --- | --- | --- | --- | --- |
|  | Internet Pretest | Pretest 2 | Internet Pretest | Pretest 2 | Pretest 3 |
|  | % correct emotion > others | % correct answers | % correct emotion > others | % correct answers | % correct congruency answers |
| Anger | 79.3 | 80.3 | 93.5 | 95.2 | 92.2 |
| Embarrassment | 84.1 | 84.7 | 88.8 | 90.4 | 85.9 |
| Pride | 94.5 | 90.1 | 86.8 | 88.6 | 94.9 |
| Neutral | 85.8 | 85.1 | 82.7 | 86.8 | 80.4 |
| Surprise | 86.9 | 86.6 | 82.1 | 84.2 | 90.0 |
| Total % | 86.1 | 85.4 | 86.8 | 89.0 | 88.7 |
| Total % Pretests 1 and 2 | 85.7 | | 87.9 | |  |

Note: The % of correct emotion > others refers to the percentage of responses for the correct emotion compared to the percentage of other emotions recognized. The % correct answers refers to the percentage of correct emotion labelling among the 5 possibilities. The % correct congruency answers refers to the percentage of correct judgment of congruency between the Reasoning and Decoding phases.

Based on these 3 pretests, we selected the best videos to fit our design.

**Table 1.**

**Figure 1.**


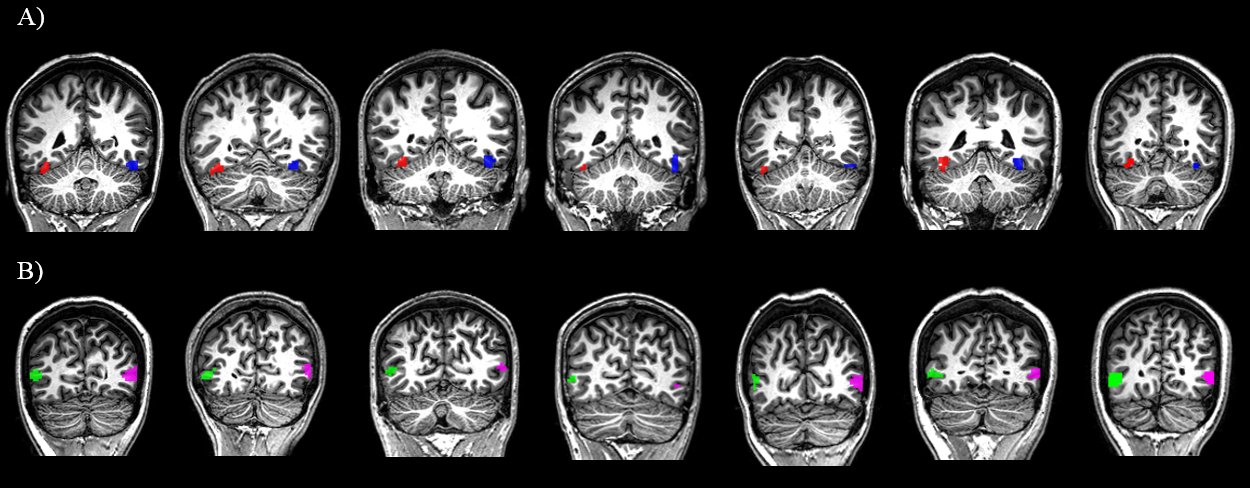


**Table 2.**

| Contrast | Region | Side | MNI Coordinates | | | T-value | Cluster Size |
| --- | --- | --- | --- | --- | --- | --- | --- |
|  |  |  | *x* | *y* | *z* |  | *(mm^3^)* |
| ***SCE > Neutral*** | |  |  |  |  |  |  |
|  | Frontal Superior Medial | R | 10 | 50 | 38 | 8.75 | 32448 |
|  | Superior Frontal Gyrus | L | -4 | 56 | 34 | 8.50 | 34624 |
|  | Temporal pole | L | -52 | 4 | -18 | 7.98 | 109888 |
|  | Temporal pole | R | 50 | 8 | -22 | 7.73 | 301184 |
|  | Supramarginal gyrus | L | -60 | -42 | 22 | 7.57 | 84288 |
|  | Inferior Frontal Gyrus | R | 56 | 30 | -2 | 6.86 | 13248 |
|  | Amygala | R | 26 | -10 | -16 | 6.74 | 11968 |
|  | Superior Frontal Gyrus | L | -12 | 0 | 72 | 6.47 | 2880 |
|  | Superior Frontal Gyrus | R | 14 | 2 | 72 | 6.45 | 2176 |
|  | Superior Occipital Gyrus | L | -12 | -88 | 32 | 6.20 | 8896 |
|  | Fusiform Gyrus | L | -42 | -44 | -20 | 6.19 | 3200 |
|  | Thalamus | L | -4 | -12 | 2 | 6.15 | 6848 |
|  | Superior Frontal Gyrus | L | -4 | 16 | 62 | 6.07 | 6272 |
|  | Amygala | L | -24 | -2 | -24 | 5.93 | 2944 |
|  | Precentral Gyrus | R | 52 | 6 | 38 | 5.79 | 3008 |
|  | Superior Temporal Gyrus | L | -58 | -32 | 0 | 5.53 | 1152 |
|  | Lingual Gyrus | L | -2 | -76 | 4 | 5.33 | 896 |
|  |  |  |  |  |  |  |  |
| ***Neutral > SCE*** | |  |  |  |  |  |  |
|  | Lingual Gyrus | L | -14 | -92 | -10 | 5.75 | 2304 |
|  |  |  |  |  |  |  |  |
| ***BE > Neutral*** | |  |  |  |  |  |  |
|  | Lateral Occipital Gyrus | R | 50 | -64 | 24 | 7.53 | 144512 |
|  | Superior Frontal Gyrus | L | -4 | 14 | 62 | 7.18 | 10624 |
|  | Frontal Pole | R | 8 | 50 | 40 | 7.12 | 24448 |
|  | Frontal Orbital Cortex | L | -44 | 28 | -6 | 7.00 | 73280 |
|  | Superior Frontal Gyrus | L | -6 | 54 | 32 | 6.94 | 22656 |
|  | Temporal Pole | L | -52 | 4 | -16 | 6.83 | 10752 |
|  | Superior Frontal Gyrus | R | 4 | 12 | 66 | 6.75 | 5952 |
|  | Supramarginal Gyrus | L | -60 | -44 | 20 | 6.72 | 29376 |
|  | Fusiform Gyrus | R | 44 | -62 | -20 | 6.70 | 20480 |
|  | Precuneus Cortex | R | 20 | -54 | 6 | 6.37 | 8384 |
|  | Cuneus | R | 12 | -86 | 34 | 6.23 | 5760 |
|  | Lingual Gyrus | R | 12 | -86 | 34 | 6.23 | 5760 |
|  | Amygdala | L | -24 | -2 | -24 | 6.01 | 2048 |
|  | Middle Temporal Gyrus | L | -60 | -32 | -2 | 5.75 | 3968 |
|  | Lateral Occipital Gyrus | R | 44 | -80 | -10 | 5.67 | 1792 |
|  | Supramarginal Gyrus | L | -58 | -50 | 34 | 5.58 | 1088 |
|  | Amygdala | R | 30 | 0 | -20 | 5.45 | 2048 |
|  |  |  |  |  |  |  |  |
| ***Neutral > BE*** | |  |  |  |  |  |  |
|  | No significant result |  |  |  |  |  |  |
|  |  |  |  |  |  |  |  |
| ***SCE > BE*** | |  |  |  |  |  |  |
|  | Supramarginal Gyrus | R | 58 | -30 | 30 | 6.45 | 4992 |
|  | Inferior Temporal Gyrus | R | 54 | -58 | -8 | 6.35 | 3328 |
|  | Superior Parietal Lobule | L | -28 | -44 | 58 | 6.26 | 3776 |
|  | Superior Parietal Lobule | R | 32 | -38 | 44 | 5.70 | 2048 |
|  |  |  |  |  |  |  |  |
| ***BE > Neutral*** | |  |  |  |  |  |  |
|  | No significant result |  |  |  |  |  |  |
|  |  |  |  |  |  |  |  |

**Table 3.**

| Contrast | Region | Side | MNI Coordinates | | | T-value | Cluster Size |
| --- | --- | --- | --- | --- | --- | --- | --- |
|  |  |  | *x* | *y* | *z* |  | *(mm^3^)* |
| ***SCE > Neutral*** | |  |  |  |  |  |  |
|  | Lateral Occipital Gyrus | R | 46 | -68 | -2 | 9.62 | 45568 |
|  | Lateral Occipital Gyrus | L | -48 | -74 | 6 | 8.28 | 24704 |
|  | Inferior Temporal Gyrus | R | 46 | -44 | -20 | 7.28 | 10560 |
|  | Middle Occipital Gyrus | R | 34 | -90 | 12 | 7.25 | 12800 |
|  | Middle Occipital Gyrus | L | -18 | -96 | 4 | 7.12 | 5376 |
|  | Fusiform Gyrus | L | -42 | -44 | -20 | 6.21 | 832 |
|  | Superior Temporal Gyrus | R | 44 | -32 | 2 | 6.08 | 2368 |
|  | Temporal Pole | R | 52 | 8 | -24 | 6.02 | 2752 |
|  | Superior Parietal Lobule | R | 24 | -54 | 56 | 5.93 | 1600 |
|  | Precentral Gyrus | L | -36 | -22 | 66 | 5.67 | 832 |
|  |  |  |  |  |  |  |  |
| ***Neutral > SCE*** | |  |  |  |  |  |  |
|  | Middle Frontal Gyrus | R | 46 | 28 | 38 | 6.71 | 9920 |
|  | Angular Gyrus | R | 56 | -50 | 50 | 6.62 | 28032 |
|  | Paracingulate Gyrus | R | 6 | 36 | 36 | 6.49 | 2624 |
|  | Frontal Pole | R | 44 | 46 | -6 | 6.29 | 6528 |
|  | Angular Gyrus | L | -44 | -56 | 48 | 5.79 | 1728 |
|  |  |  |  |  |  |  |  |
| ***BE > Neutral*** | |  |  |  |  |  |  |
|  | Lateral Occipital Gyrus | L | -50 | -66 | 10 | 7.82 | 42752 |
|  | Superior Temporal Gyrus | L | -54 | -14 | -8 | 7.53 | 30848 |
|  | Lateral Occipital Gyrus | R | 44 | -64 | 4 | 7.39 | 25280 |
|  | Superior Temporal Gyrus | R | 58 | -8 | -10 | 7.33 | 59456 |
|  | Fusiform Gyrus | L | -42 | -44 | -20 | 7.30 | 5440 |
|  | Inferior Occipital Gyrus | R | 36 | -80 | -10 | 6.41 | 2624 |
|  | Fusiform Gyrus | R | 42 | -56 | -14 | 6.38 | 4160 |
|  | Lingual Gyrus | L | -4 | -84 | -10 | 6.05 | 3328 |
|  | Fusiform Gyrus | L | -18 | -80 | -10 | 5.64 | 1344 |
|  |  |  |  |  |  |  |  |
| ***Neutral > BE*** | |  |  |  |  |  |  |
|  | Frontal Pole | R | 40 | 48 | -8 | 6.96 | 12416 |
|  | Angular Gyrus | R | 56 | -50 | 50 | 6.58 | 18944 |
|  | Frontal Superior Gyrus | R | 22 | 28 | 48 | 5.69 | 3904 |
|  |  |  |  |  |  |  |  |
| ***SCE > BE*** | |  |  |  |  |  |  |
|  | Middle Occipital Gyrus | L | -20 | -96 | 2 | 6.75 | 1984 |
|  |  |  |  |  |  |  |  |
| ***BE > SCE*** | |  |  |  |  |  |  |
|  | Superior Temporal Gyrus | L | -58 | -22 | -6 | 5.86 | 4096 |
|  | Lingual Gyrus | L | -6 | -80 | -8 | 5.66 | 1408 |
|  |  |  |  |  |  |  |  |
|  |  |  |  |  |  |  |  |
